# Spectrophotometric Assays for Sensing Tyrosinase Activity and Their Applications

**DOI:** 10.3390/bios11080290

**Published:** 2021-08-23

**Authors:** Yu-Fan Fan, Si-Xing Zhu, Fan-Bin Hou, Dong-Fang Zhao, Qiu-Sha Pan, Yan-Wei Xiang, Xing-Kai Qian, Guang-Bo Ge, Ping Wang

**Affiliations:** 1 Shanghai Frontiers Science Center for Chinese Medicine Chemical Biology, Institute of Interdisciplinary Integrative Medicine Research, Shanghai University of Traditional Chinese Medicine, Shanghai 201203, China; fyfshutcm@163.com (Y.-F.F.); hfbshutcm@163.com (F.-B.H.); zdf9615@163.com (D.-F.Z.); pansha@shutcm.edu.cn (Q.-S.P.); qxkgood@hotmail.com (X.-K.Q.); geguangbo@shutcm.edu.cn (G.-B.G.); 2 Institute of Science, Technology and Humanities, Shanghai University of Traditional Chinese Medicine, Shanghai 201203, China; zhusx_tcm@163.com; 3 School of Rehabilitation Science, Shanghai University of Traditional Chinese Medicine, Shanghai 201203, China; xiangsunkey@163.com

**Keywords:** tyrosinase (TYR), enzymatic activity, optical substrates, TYR inhibitors, high-throughput screening

## Abstract

Tyrosinase (TYR, E.C. 1.14.18.1), a critical enzyme participating in melanogenesis, catalyzes the first two steps in melanin biosynthesis including the *ortho*-hydroxylation of L-tyrosine and the oxidation of L-DOPA. Previous pharmacological investigations have revealed that an abnormal level of TYR is tightly associated with various dermatoses, including albinism, age spots, and malignant melanoma. TYR inhibitors can partially block the formation of pigment, which are always used for improving skin tone and treating dermatoses. The practical and reliable assays for monitoring TYR activity levels are very useful for both disease diagnosis and drug discovery. This review comprehensively summarizes structural and enzymatic characteristics, catalytic mechanism and substrate preference of TYR, as well as the recent advances in biochemical assays for sensing TYR activity and their biomedical applications. The design strategies of various TYR substrates, alongside with several lists of all reported biochemical assays for sensing TYR including analytical conditions and kinetic parameters, are presented for the first time. Additionally, the biomedical applications and future perspectives of these optical assays are also highlighted. The information and knowledge presented in this review offer a group of practical and reliable assays and imaging tools for sensing TYR activities in complex biological systems, which strongly facilitates high-throughput screening TYR inhibitors and further investigations on the relevance of TYR to human diseases.

## 1. Introduction

**Figure 1:**
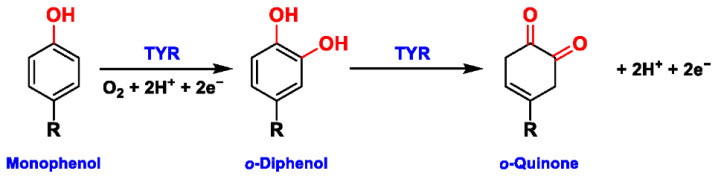
The slow *ortho*-hydroxylation of monophenol and the fast oxidation of catechol catalyzed by TYR.

**Figure 2:**
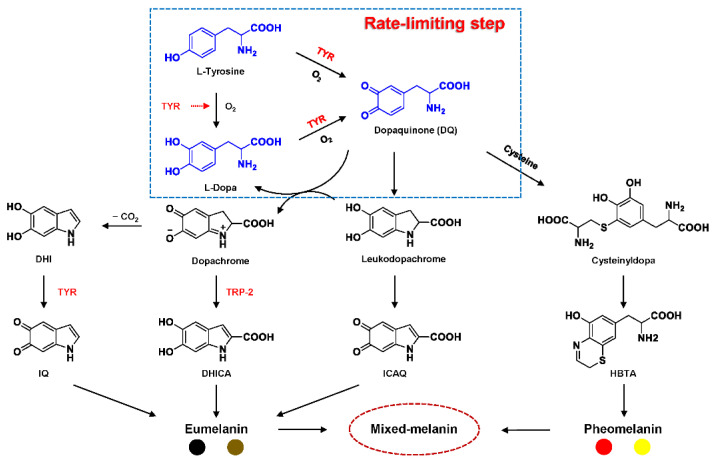
Biosynthetic pathway of melanin [7,8,10]. DQ: dopaquinone; L-Dopa: L-3,4-dihydroxyphenylalanine; DHICA: 5,6-dihydroxyindole-2 carboxylic acid; DHI: 5,6-dihydroxyindole; ICAQ: indole-2-carboxylic acid-5,6-quinone; IQ: indole-5,6-quinone; HBTA: 5-hydroxy-1,4-benzothiazinylalanine.

Tyrosinase (TYR, E.C. 1.14.18.1), a type-3 binuclear copper-containing oxidoreductase, efficiently catalyzes *o*-hydroxylation of monophenols to diphenols (monophenolase activity) and the oxidation of diphenols to quinones (diphenolase activity), without any additional cofactors (Figure 1) [1,2]. It is ubiquitously distributed in organisms ranging from bacteria to eukaryotes and plays a pivotal role in the enzymatic browning of fruit or fungi, as well as mammalian melanin synthesis [3,4]. In mammals, melanin is exclusively synthesized in melanosomes via complex biochemical reactions (Figure 2), and this endogenous substance is primarily responsible for the pigmentation of retina and skin [5,6]. TYR catalyzes the first two steps in melanin biosynthesis: the *o*-hydroxylation of L-tyrosine and the oxidation of L-DOPA. Since the remainder of the reaction sequence can proceed spontaneously at physiological pH, the conversion of L-tyrosine to dopaquinone (DQ) has been implicated as a crucial rate-limiting procedure in melanogenesis [7,8]. DQ could spontaneously convert into dopachrome, which gradually decomposes into 5,6-dihydroxyindole (DHI) and 5,6-dihydroxyindole-2-carboxylic acid (DHICA) through a succession of redox reactions [5,9]. Ultimately, these dihydroxyindoles are oxidized to eumelanin. Alongside, in the presence of cysteine or glutathione, DQ is converted to 5-*S*-cysteinyl dopa or glutothionyl dopa, finally yielding pheomelanin [10,11]. The types and relative amounts of these melanin constitute color-based ethnic diversification. Three tyrosinase-like enzymes co-regulate melanogenesis, including TYR and TYR-related proteins 1 (TRP-1) and 2 (TRP-2). TRP-1 shows DHICA oxidase and low tyrosine hydroxylase activity when zinc is replaced by copper. TRP-2 contains two zinc ions at the active site and isomerizes dopachrome to DHICA. They are metal-containing glycoproteins and share ~40% amino acid sequence identity and ~70% similarity [1]. Despite TYR and TYR-related proteins 1 (TRP-1) and 2 (TRP-2) being necessary for melanogenesis, TYR is the most critical rate-limiting enzyme [10].

Recent pharmacological investigations have revealed that the abnormal metabolism of melanocytes and imbalance in TYR activity are indirectly or directly responsible for various dermatoses [1,12,13]. For example, functional mutations in the gene encoding TYR (TYR, 11q14-21, MIM 606933) would inactivate TYR and cause a deficiency in melanin, thereby resulting in oculocutaneous albinism type 1 (OCA1, MIM 203100), an autosomal recessive disorder characterized by the absence of pigment in hair, skin, and eyes [14]. On the contrary, excess melanin accumulation or abnormal distribution would give rise to hyperpigmentation disorders, including age spots, post-inflammatory hyperpigmentation, and even malignant melanoma [13,15]. In particular, the overexpression of TYR and TRP 1 is significantly associated with the risk of melanoma, a fatal skin carcinoma [16,17,18]. To this end, it has been viewed as a relatively specific biomarker and therapeutic target for melanoma lesions. Additionally, the abnormal level of TYR induces dopamine neurotoxicity and neurodegeneration, which is related to Parkinson’s disease (PD) [19,20,21]. Monitoring TYR activity in complex biosystems undoubtedly remains critical and challenging for biomedical research and drug high-throughput screening (HTS).

In recent decades, various analytical techniques, including immunochemical analysis, mass spectrometry-based proteomics, and substrate-based biochemical assays, have flourished for the quantification of TYR. However, only the substrate-based biochemical assay could rapidly and sensitively determine the real activity of TYR in complex biological systems, leading to its common use in drug discovery and clinical studies [22]. Herein, we will review the role of biochemical detection based on optical substrates in TYR detection in the past 40 years. This review covers breakthroughs in the development of probe substrates and corresponding analytical methodologies for sensing TYR activity. By listing the substrate information and kinetic parameters of several optical methods for the first time, the advantages and defects of these various approaches are sufficiently compared and analyzed. Ultimately, the challenges and future perspectives in this field are highlighted. Collectively, this review provides a practical reference for developing new TYR substrates and methods, which is of great significance for related diseases studies and medical screening.

## 2. Biochemical Characteristics of TYR

### 2.1. Structural Feature and Catalytic Mechanism of TYR

**Figure 3:**
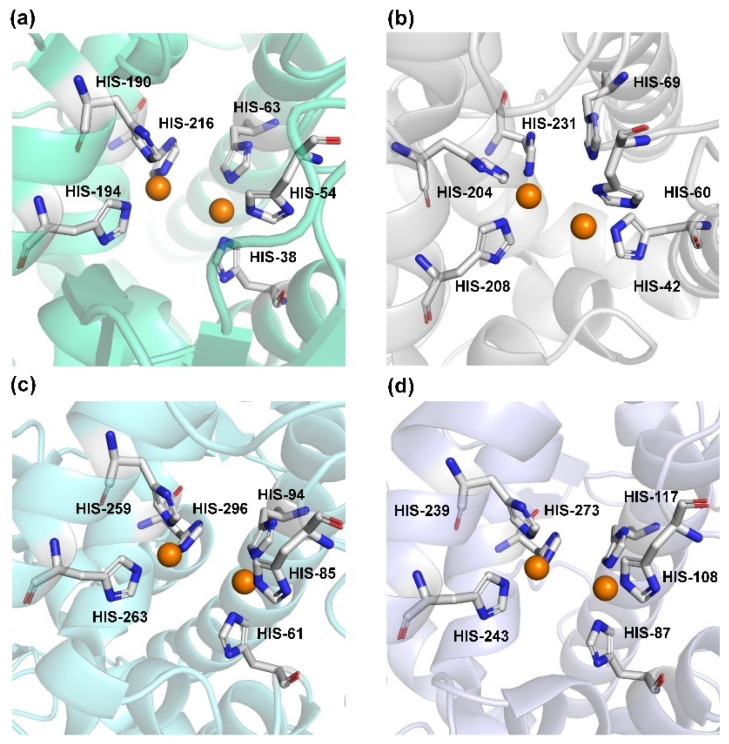
The conserved cavity of TYR from different sources. (**a**) The crystal of TYR from *Streptomyces castaneoglobisporus* (PDB ID: 2ZMX). (**b**) The crystal of TYR from *Bacillus megaterium* (PDB ID: 3NQ1). (**c**) The Crystal of TYR from fungus (PDB ID: 2Y9W, *Agaricus bisporus*). (**d**) The Crystal of TYR from plant (PDB ID: 5CE9, *Juglans regia*). Two copper ions (orange) are coordinated with three histidine residues, respectively.

**Figure 4:**
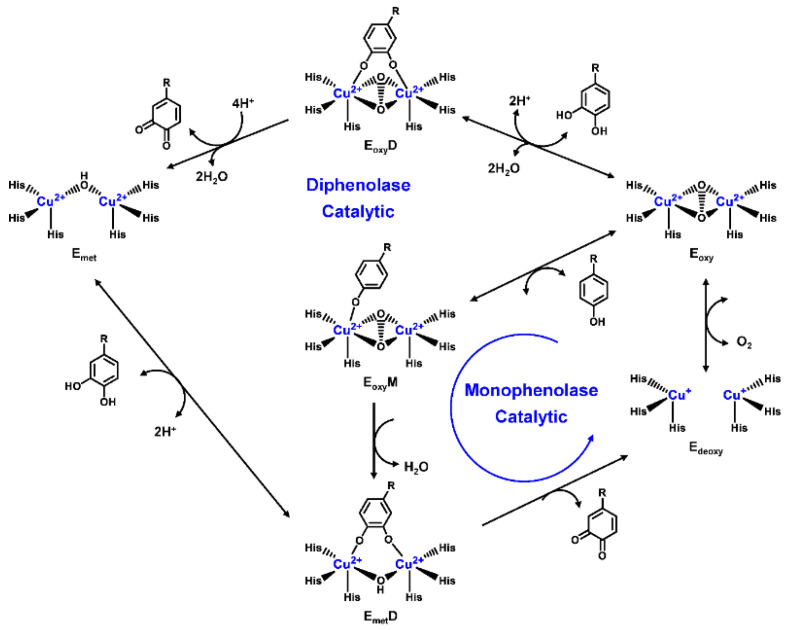
Catalytic cycle of TYR.

Human TYR is a glycoprotein (13% carbohydrate) predominantly located in the melanosome membrane of melanocytes [23,24]. The presence of the transmembrane domain and glycans renders it difficult to isolate homogeneous TYR from melanocytes, which impedes crystallographic studies. Fortunately, the most characteristic TYR can be acquired from *Streptomyces glausescens*, the fungi *Neurospore crassa*, or *Agaricus bisporus* [7,25,26]. To compare the conservation of catalytic cavity, the crystal structures of TYR from bacteria (*Streptomyces castaneoglobisporus* [25] and *Bacillus megaterium* [27]), fungi *Agaricus bisporus* [28], and walnut leaves [29] were retrieved from Protein Date Bank. A salient feature of TYR from various sources (Figure 3) is the presence of strictly conserved binuclear copper atoms at the active site, each of copper atoms is coordinated with three conserved histidines, respectively [3]. Moreover, the normal redox state of copper atoms is exceedingly significant for enzyme activity. Due to the relatively high similarity and homology with mammalian TYR, mushroom TYR from *Agaricus bisporus* acts as a model for enzyme kinetics and inhibitor screening [28,30,31]. Although extensive studies are devoted to TYR, its catalytic mechanism remains controversial. According to the presence/absence of oxygen and the oxidation state of copper ions [Cu (Ⅱ)/Cu (Ⅰ)], three enzymatic forms (E_oxy_, E_met_, and E_deoxy_) participate in the catalytic cycle (Figure 4) [3,32]. The resting form of TYR is found to be a mixture of 85% met and 15% oxy forms, while only the latter could act on the monophenol [7,33,34]. During the monophenolase cycle, to form E_oxy_-monophenol complex (E_oxy_M), the oxygen atom on the deprotonated monophenol is coordinated with the coppers of E_oxy_. Then, the phenol is *o*-hydroxylated to generate E_met_-diphenol (D) complex (E_met_D) [26,35]. Reducing agents could well draw the E_met_ into the E_deoxy_, with concomitant oxidation to the corresponding *o*-quinone [36]. Since the deoxy form is the only one capable of reacting with oxygen to regenerate E_oxy_ and continue the catalytic action, monophenolase activity usually manifests as a characteristic lag time until a sufficient amount of catechol helps E_met_ to become E_deoxy_ [36]. Remarkably, this period depends on several factors, including enzyme concentration, monophenol concentration and the presence of reducing agents, especially *o*-diphenol derivatives (such as L-DOPA) that could shorten and even abolish the lag time [30,33,37]. In the diphenolase cycle, E_oxy_ continues to bind *o*-diphenol to originate the E_oxy_D complex, while both E_oxy_ and E_met_ are capable of oxidizing the diphenol to the *o*-quinone. After this, E_met_ is regenerated to complete the catalytic cycle continuously [30,34].

### 2.2. Substrate Specificity of TYR

**Figure 5:**
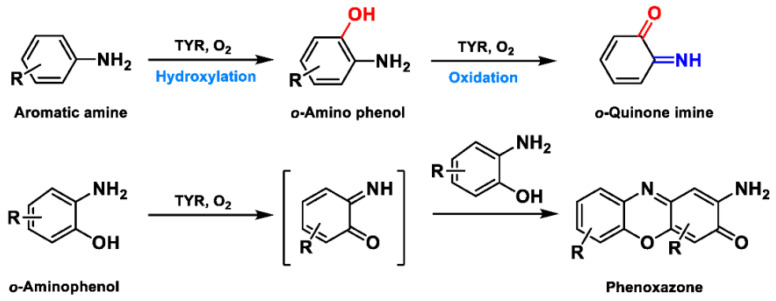
The catalytic reaction of TYR-mediated aromatic amine and *o*-aminophenol. Adapted with permission from ref. [43]. 1987, American Chemical Society.

Based on the broad substrate spectrum, in principle, any simple monophenol or corresponding catechol appears to be its substrate [38]. Besides, TYR also oxidizes various aromatic amines, *o*-aminophenols, and aromatic *o*-diamines (Figure 5), despite the reaction rates being orders of magnitude smaller than the corresponding phenols or catechol [39,40]. In terms of phenols, mammalian TYR tends to be relatively specific for its physiological substrate (L-tyrosine and L-DOPA) and has a higher affinity for the L-isomers [41]. A prevalent characteristic in monophenol substrates is without substituents in the *ortho*-position of the phenolic hydroxyl group. Understandably, large side-chain substituents increase the difficulty of substrate interaction with the key catalytic residues; this is unpropitious for the recognition and catalytic process between the enzyme and ligand [36]. A kinetic study [42] quantitatively discussed the effects of substituents in the 1-position of the aromatic ring on the rate of hydroxylation catalyzed by TYR. The results revealed that monophenols with a high electron donor tend to be oxidized faster [42]. In sharp contrast, the oxidation rate of catechol is positively correlated with the electron-withdrawing capacity of the *para*-substituents [36]. As such, the steric hindrance, stereochemical characteristics, and electronic effects of substituents have a distinct influence on the rate of TYR-mediated catalysis.

According to the chemical stability of the corresponding *o*-benzoquinone, phenolic substrates (Table 1) can be roughly divided into the following three categories [42,44]. (1) The first sort of substrates catalyzed by TYR could yield stable *o*-quinones. For example, 4-*tert*-butylcatechol (TBC) is detectable for the diphenolase activity, whose *o*-quinone is exceedingly stable. (2) The second substrates produce a highly unstable *o*-quinone but evolve into a stable product via a first-order reaction. Targeted at diphenolase activity, the common determination is based on the formation of dopachrome using L-DOPA as a substrate. Moreover, there is 3,4-dihydroxymandelic acid (DOMA) [45], dopamine (DA) [46], and isoproterenol (ISO) [47]. (3) The third kind of substrates are oxidized to an unstable *o*-quinone that is vulnerable to potent nucleophiles (N) and yields chromatic adducts (NQ) with a clear stoichiometry. Commonly used nucleophiles include L-proline (Pro) [48], L-cysteine (Cys) [49], and especially 3-methyl-2-benzothiazolinone hydrazone (MBTH) [42]. Related substrates mainly include 4-hydroxyphenylacetic acid, 4-hydroxyphenylpropionic acid, L-DOPA, DA, etc.

## 3. Optical Assays for Sensing TYR Activity

The development of analytical techniques mainly focuses on improving and monitoring bioactive species, especially real-time analysis in vivo [50,51,52,53]. The current optical methods, including spectrophotometry and fluorometric detection, exhibit distinct performance. In this paper, we review the research advances of various methods by emphasizing on both their pros and cons, and we also summarize the non-fluorescent substrates and fluorescent substrates.

### 3.1. Spectrophotometric Assays

Due to its intrinsic sensitivity, low cost, and continuous study of the reaction process, the spectrophotometric technique has become the most widely used method [44,54]. Hitherto, in vitro assays of the oxidase activity of TYR are predominantly comprised of the dopachrome formation methods that use L-tyrosine or L-DOPA as substrate [55,56,57,58]. However, this mainstream approach also has inherent flaws. It relies on the hypothesis that DQ (an oxidation product of L-DOPA) is completely converted to dopachrome, instead of directly measuring DQ [57]. The instability and relatively low absorption coefficient of dopachrome in an aqueous system mean that the test must be performed quickly and at a low sensitivity. Considering the high reactivity and instability of intermediates and the interference of external factors (such as temperature and oxygen), many other assays are subsequently improved. Many nucleophiles start appearing on the stage by capturing highly reactive DQ as stable-colored products [59]. Under acidic conditions, MBTH generate a pink adduct with high molar absorptivity and solubility, whose clear stoichiometry and high stability endow great measurability [42,44,60].

Distinct from the above, the quinonization product of 4-tert-butylcatechol (TBC) is remarkably stable, simple to accumulate in the reaction mixture, and facile to detect. Lamentably, its affinity (*K_m_* = 990 μM) toward TYR is exceedingly poor, which restricts its application [37]. More typical substrates and their optical parameters are documented in Table 2.

**Figure 6:**
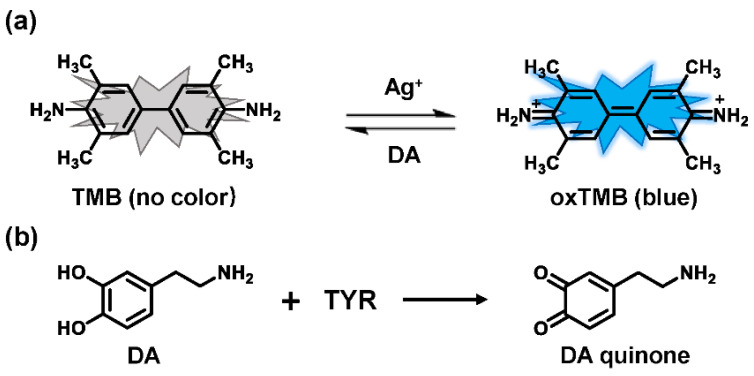
Detection system of Ag^+^-TMB with dopamine as substrate. (**a**) Mechanism of the colorimetric assay based on Ag^+^-TMB system for TYR activity. (**b**) The metabolic reaction of dopamine catalyzed by TYR.

In recent years, some breakthroughs have been made in substrates identification and detection means. Several sensing platforms utilized the reducibility of catechol to capture some chromogenic reagents for quantitative analysis [62,63]. For instance, Ag^+^ could oxidize 3,3′,5,5′-tetramethylbenzidine (TMB) to the oxidized 3,3′,5,5′-tetramethylbenzidine (oxTMB), accompanied by remarkable changes in color and absorbance [64,65]. The introduction of reducing substances (such as DA) directly decreases oxTMB, resulting in faded blue and a decrease in absorbance (Figure 6). Accordingly, using TMB as a chromogenic probe, a facile colorimetric assay was proposed to sense TYR activity in human serum samples and to screen inhibitors [66]. In a similar vein, Deng et al. demonstrated that catechol could suppress the activity of oxidase-mimicking chitosan-stabilized platinum nanoparticles (ChPtNPs), thereby significantly decreasing acidified TMB products [67]. With the oxidation of catechol, a linear relationship between the amount of restored color and the TYR activity was evaluated. In terms of detection means, inspired by specific chromogenic and fluorogenic reactions between resorcinol and catecholamines [68], an absorbance–fluorescence dual-readout assay was established. With tyrosine as a substrate, this assay achieved the determination of TYR in serum samples and inhibitor screening [69]. Notably, these innovative assays are consistent with the L-DOPA oxidation-based method.

As a classical analytical method, the spectrophotometry assay still exhibits promising prospects for in situ quantitative analysis. Furthermore, when establishing an experimental methodology, the enzyme activity in the presence of substrates, absorption coefficient or stability of products, anti-interference and sensitivity, incubation time, and even reagent consumption, ought to be taken into account [54,67].

### 3.2. Fluorometric Assays

Despite spectrophotometry being commonly used for the detection of TYR activities in vitro, this method is insufficient when it comes to high-throughput screening (HTS) or dynamic tracking [70]. Owing to their superior sensitivity, ultrahigh spatiotemporal resolution, and without isolation and derivative, the fluorescent substrate-based techniques have shown unprecedented developments in real-time visualizing and detecting biomolecules in vitro or in vivo [71,72,73]. To date, a great number of TYR-activated fluorescent substrates have flourished, primarily including organic fluorescent molecules and nanometer material-based probes [74].

#### 3.2.1. Small Molecule-Based Fluorescent Substrates

**Figure 7:**
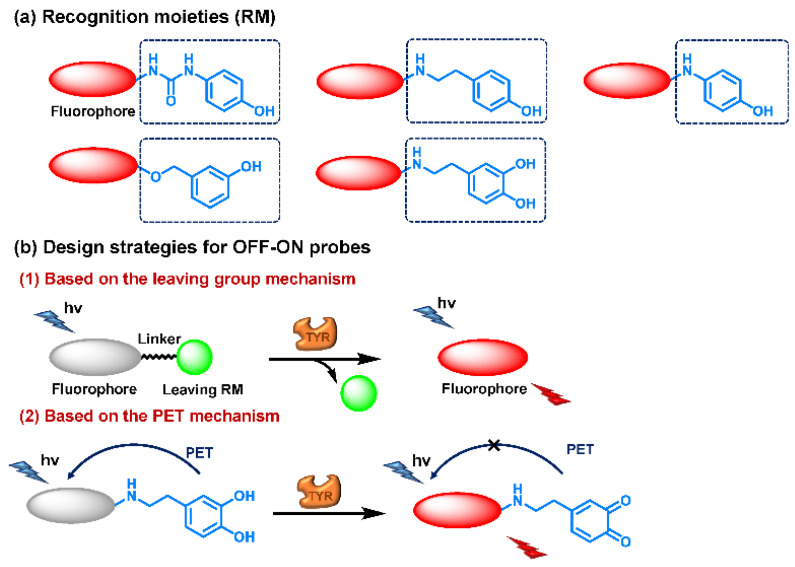
Several strategies reported for designing the fluorescent substrates of TYR. (**a**) Some typical warheads of fluorescent probe for TYR detection. (**b**) Two sensing mechanisms of OFF-ON probes towards TYR.

The current probes are mainly designed by the specific phenolic substrates (recognition moiety) in conjugation with fluorophore scaffolds through an appropriate linker [75,76]. Given the metabolic characteristics of TYR, the recognized fragment should contain a phenolic hydroxyl, without substituents in the *ortho* position; this facilitates the formation of catechol [77]. To the best of our knowledge, 4-hydroxyphenyl and 3-hydroxyphenyl are the two main types of responsive unit (Figure 7a). However, 4-hydroxyphenyl, as a classical responsive moiety for TYR, could react with both reactive oxygen species (ROS) and TYR in most cases [70]. Owing to H_2_O_2_, HOCI and some free radicals are usually at a relatively high concentration (about μM levels) especially in tumor cells; this cross-interference may result in false-positive signals and inaccurate results [78,79]. Rejoicingly, the replacement of 4-hydroxyphenyl with 3-hydroxyphenyl not only preserves binding affinity towards TYR but also avoids the influence of cross-interference from ROS. Mainstream designs primarily include the oxidization-cleavage mechanism and the inhibited photo-induced electron transfer (PET) process (Figure 7b) [80]. The former is oxidized to an unstable *o*-quinone in presence of oxygen and TYR, which undergoes an intramolecular 1,6-rearrangement-elimination, further releasing free fluorophore and triggering a fluorescence response. In the latter, the initial hydroxyphenyl group exerts a PET effect on the parent. Accompanied by the formation of *o*-quinone and the blocked PET effect, the probe is lit up. By rationally adjusting the TYR-recognition unit and fluorophore structure, a variety of probes could be acquired. The newly developed TYR fluorescent substrates and their biological parameters are presented in Table 3.

##### 4-Hydroxyphenyl Recognition Units

**Figure 8:**
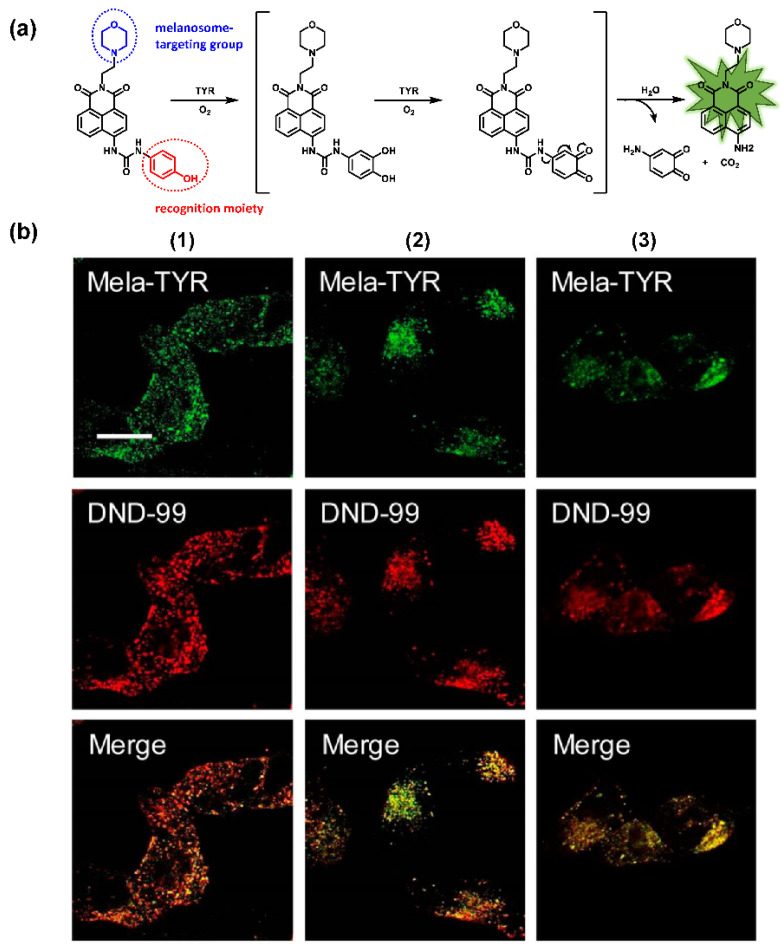
(**a**) The structure of Mela-TYR and its reaction mechanism with TYR. (**b**) Colocalization of Mela-TYR and lysosome tracker DND-99 in B16 cells. The cells were pretreated with inulavosin (10 μM) for (1) 0, (2) 6, and (3) 12 h, respectively, and then incubated with Mela-TYR and DND-99 [89]. Copyright permission is granted by American Chemical Society.

**Figure 9:**
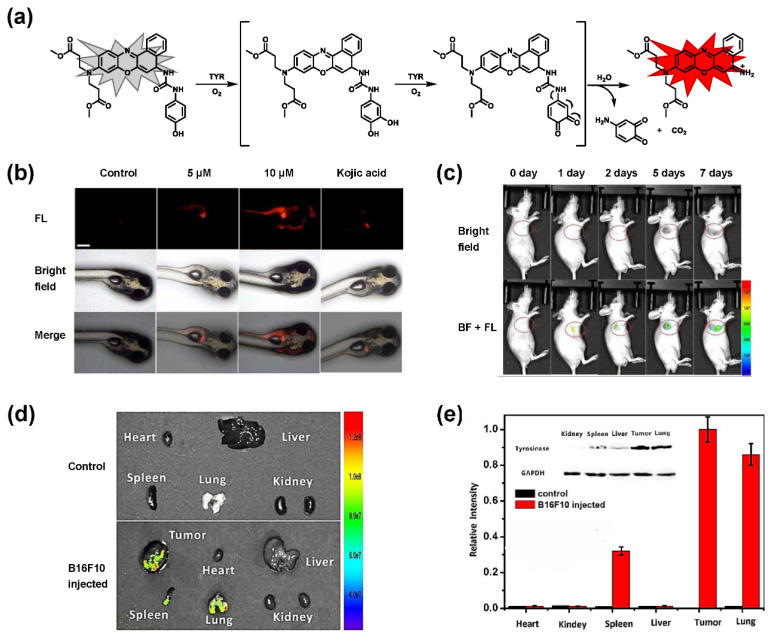
(**a**) The schematic diagram of NBR-AP for TYR detection. (**b**) Images for 3-day-old zebrafish incubated with various concentrations of the probe and kojic acid. (**c**) Fluorescent and bright field images of 4-week-old mice injected with the probe upon injection with B16F10 cells. (**d**) Images of dissected organs of the mice injected with the probe upon injection with B16F10 cells for 14 days. (**e**) Relative intensity values (n = 3) obtained from (**d**) and calculated using Image J2x software, while the relative intensity from tumor is defined as 1.0 [88]. Copyright permission is granted by American Chemical Society.

The classic structure of 4-hydroxyphenyl is covalently coupled with fluorophore via a carbamate linkage. A novel probe Mela-TYR with melanosome-targeting ability first imaged the distribution of TYR in organelles (Figure 8) [89]. This probe utilized morpholine as a melanosome-targeting group and 4-aminophenol as a responsive warhead. Since the acidic environment (about pH 4.2–4.6) of melanosomes, the protonated form of morpholine enhanced the hydrophilicity of morpholine and facilitated its accumulation in melanosomes. Subsequently, the colocalization experiments with mCherry-tagged melanosomes and DND-99 (a commercial dye) validated this targeting ability. Through Mela-TYR imaging, it was found that TYR was significantly up-regulated in live B16 cells stimulated by psoralen/ultraviolet light A, which was further verified by standard colorimetric methods. To observe another representative example, the near-infrared (NIR) probe NBR-AP was activated through an oxidization-cleavage reaction and displayed a linear relationship over the range of 1~200 U/L [88]. Moreover, NBR-AP accomplished the sensitive and selective detection of endogenous TYR activity in B16F10 cells and zebrafish (Figure 9). Real-time in vivo imaging of melanoma and metastasis in xenogeneic mouse models suggested that NBR-AP may be a reliable approach for the early diagnosis of metastatic melanoma during cancer surgery. To the best of our knowledge, this was the first investigation to utilize a fluorescent substrate for the diagnosis of early melanoma in a rodent model. Follow-up studies demonstrated that a longer linker between the recognition moiety and the dye skeleton might prominently decrease the steric hindrance of the probe entering the catalytic site. By combining thermodynamic computation with molecular docking simulation, Li et al. analyzed the Gibbs free energy change of different urea bonds during spontaneous hydrolysis, as well as the distance between the phenolic hydroxyl group (metabolic site) and the catalytic site [92]. Thus, a rapidly responsive and ultra-sensitive NIR probe MB1 was rationally designed. As a specific substrate of TYR (*K_m_* = 4.6 µM; *V_max_* = 0.45 µM/min), the fluorescence intensity of MB1 could increase >100-fold within 20 min, providing immense convenience for drug screening. Notably, this sensor could effectively kill melanoma cells by photodynamic therapy (PDT). As such, this sensor held great potential in melanoma-specific imaging and treatment.

##### 3-Hydroxyphenyl Recognition Units

**Figure 10:**
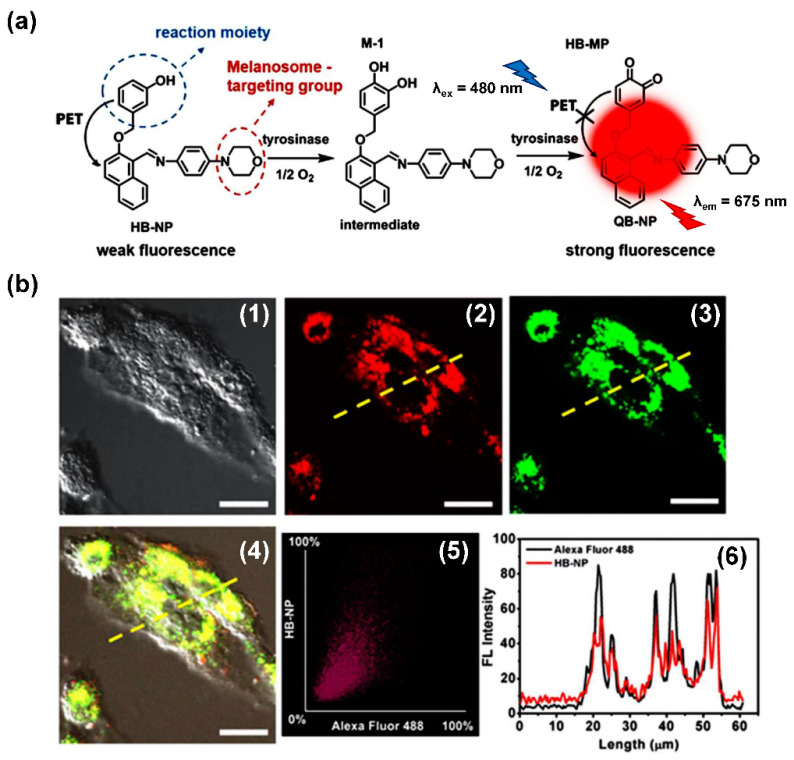
(**a**) The mechanism of TYR activity detected by HB-NP. (**b**) Colocalization of HB-NP and Alexa Fluor 488 (a commercial dye) in B16 cells [80]. (1) Bright-field image of the B16 cells; (2) fluorescence image of the red channel for HB-NP; (3) fluorescence image of the green channel for Alexa Fluor 488 (λ_ex_ = 488 nm, λ_em_ = 500–555 nm); (4) the merged image of (1)–(3). (4) Intensity correlation plot of HB-NP and Alexa Fluor 488. (6) Intensity profile of the linear ROI across the cell (yellow line in images (2)–(4)). Copyright permission is granted by American Chemical Society.

When ROS reacts with the hydroxyl recognition unit, it tends to form quinone derivatives rather than hydroxylated product. On this premise, to eliminate the interference from ROS, 3-hydroxyphenyl is proposed. Wu et al. developed NIR probe 1. This sensor displayed a specific response to TYR, even when the concentration of ROS was much higher than that found at physiological levels. The high specificity of probe 1 facilitated the accurate detection of TYR activity in live cells and zebrafish, which was further verified by ELISA [82]. Inspired by the excellent work of predecessors, Peng et al. constructed a NIR melanosome-targeting probe (HB-NP) for the highly selective detection of TYR at the subcellular level (Figure 10) by incorporating 3-hydroxyphenyl moiety and the morpholine unit (melanosome-targeting group) into the salicyladazine skeleton [80]. Compared to Mela-TYR, the probe exhibited a large Stokes shift (195 nm) after PET effect inhibition. HB-NP successfully visualized and quantified intracellular TYR activity in various living cells. Moreover, HB-NP distinguished two human uveal melanoma cells with different invasive behaviors and evaluated the effects of the inhibitor (kojic acid) and the up-regulating treatment (psoralen/ultraviolet A). Zhang et al. designed a novel, water-soluble probe that detected the endogenous TYR in living cells and zebrafish [83]. The recognition fragment, 3-hydroxybenzyloxy, could specifically identify TYR instead of ROS. In particular, the probe successfully realized the diagnosis of melanoma in a xenogeneic mouse model.

In brief, each of the above probes exhibits unique performance. For high-throughput screening at the target level in vitro, the sensitivity and rapid response of tool molecular demonstrate more importance [96]. On the other hand, the tracing and visualization analysis of TYR activity in vivo requires long emission wavelengths and specificity [97,98]. In the near future, combining computational means such as molecular docking, more superior sensors for various purposes are expected to be rationally designed; these sensors have broad application potential in the field of bioanalysis.

#### 3.2.2. Nanoparticle-Based Fluorescent Probes

**Figure 11:**
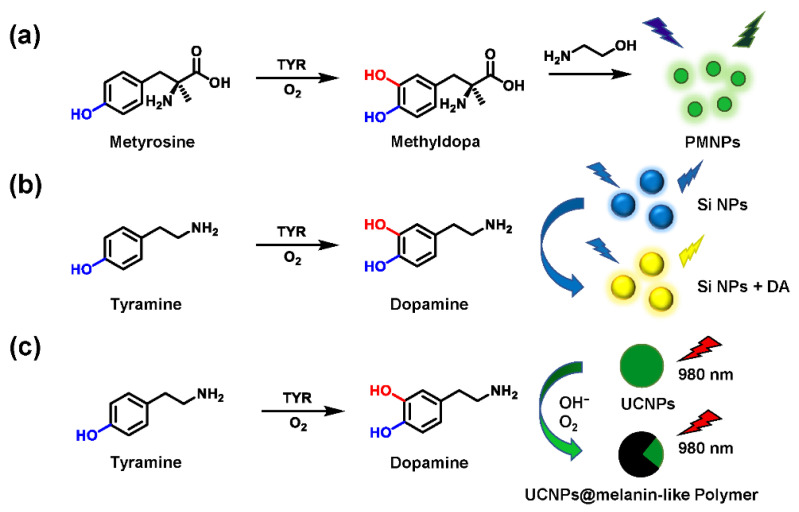
Schematic illustration of the PMNPs (**a**), Si NPs (**b**), and UCNPs (**c**) for the TYR activity detection.

Other than small molecular probes, nanometer material-based biosensors have also aroused considerable interest in the detection of biological analytes. Some emerging nanomaterials utilize the characteristics of TYR-mediated metabolism to trigger a linear fluorescence response with intermediates. For example, under aerobic and alkaline conditions, dopamine is converted to polydopamine via oxidation and self-polymerization. Enlightened by the intrinsic fluorescence properties of polydopamine, Liu et al. prepared the fluorescent polymethyldopa nanoparticles (PMNPs) [99]. Metyrosine acted as a substrate of TYR to yield methyldopa (a dopamine analog). The latter further reacted with ethanolamine to produce PMNPs (Figure 11a). Ultimately, the strategy of in situ formation of fluorescent PMNPs performed well in screening inhibitors. Using a similar principle, Ding et al. introduced tyramine as a model substrate, which could be converted into DA by TYR. Based on the specific sensing between silicon nanoparticles (Si NPs) and DA, the solution color and fluorescence emission changed significantly (Figure 11b). Subsequently, a novel ratiometric fluorescence analysis was established for screening TYR activators and inhibitors [100]. Wang et al. developed a fluorescence-sensing platform utilizing rare-earth-doped upconversion nanoparticles (UCNPs) [101]. Tyramine was oxidized to DA and further yielded melanin-like polymers, leading to the effective quenching of UCNPs (Figure 11c). Collectively, most nanoparticle-based fluorescent probes have fulfilled relevant drug screening protocols. Some potential factors, including complicated synthesis, time-consuming sample pretreatment, homogeneity, and stability of nanoparticles, deserve adequate consideration. Furthermore, compared to traditional colorimetry, both methods seem to be suitable only for TYR activity evaluation in vitro.

## 4. Biomedical Applications of TYR Activity Assays

### 4.1. Sensing and Imaging TYR Activities in Biological Systems

Melanoma is the most aggressive malignancy in skin cancer. It is characterized by high metastatic potential, poor prognosis, and the up-regulation of melanocytes [102]. Reintgen et al. determined the order of melanoma nodal metastases and showed that cutaneous melanoma usually first metastasized to the regional nodal basin via the regional lymphatics [103,104]. About one-third of melanoma patients have a local recurrence, while distant metastasis at the initial site of relapse is also relatively common [105]. The most characteristic metastatic sites are lymph nodes, lungs, liver, brain, and bones [106]. The precise detection of subclinical metastases for early diagnosis and treatment of melanoma is a matter of urgency. Human TYR (11q14-21, MIM 606933) is primarily expressed in epidermal, follicular, and ocular melanocytes. It is essential for pigment formation [107]. The overexpression of TYR and TRP 1 is significantly associated with the risk of melanoma [1]. B16F10 cells are often selected as cell models due to the high TYR expression. TYR has acted as a valuable tumor marker and therapeutic target for the early diagnosis and treatment of melanoma lesions. As a critical melanoma-associated antigen, TYR can be recognized by autologous T lymphocytes, thus inducing effective tumor-specific responses.

Currently, a serum assay remains the mainstream approach for biomarker detection [88]. Reverse transcription-polymerase chain reaction (RT-PCR) analysis was developed to detect circulating melanoma cells (CMCs) in the peripheral blood. This method was based on the amplification of the messenger RNA (mRNA) for TYR, while normal melanocytes are not thought to circulate in the peripheral blood [108,109,110]. However, test results are often controversial and can show false positives; the latter may be due to sample processing and the transient presence of metastasizing tumor cells [111]. By adopting in vivo fluorescent imaging strategy, the elevation of the TYR level (or activity) at the melanoma focus can be localized spatially, thereby lessening the risk of false-positive signals. The NIR probe NBR-AP successfully realized the early diagnosis of melanoma and metastasis in a mouse model by imaging TYR activity [88]. The levels of TYR in the tumor and metastatic organs analyzed by Western blot were consistent with the fluorescence results (Figure 9e), in which TYR in the tumor, lung, and spleen was found to be over-expressed. These results demonstrate that small-molecule fluorescent probes have great promise in the early diagnosis of melanoma and the analysis of biological samples.

### 4.2. Screening and Characterization of TYR Inhibitors

TYR is the initiating and rate-limiting factor in melanin biosynthesis, serving as a prominent target for pigmentation disorders [9,10,112,113]. Accordingly, TYR inhibitors can block the formation of pigment and exhibit broad application prospects in agricultural, medicinal, and cosmetic industries [114]. Most compounds are reported as TYR inhibitors due to their function in copper chelation or competition with substrates, while the former could give rise to the irreversible inactivation of TYR [26,36,115].Popular whitening agents, such as hydroquinone, β-arbutin (a hydroquinone derivative), or kojic acid, have always been regarded as positive controls, but they also have certain drawbacks (Table 4). Efficiently discovering potent TYR inhibitors with superior safety profiles remains a mainstream concern in hyperpigmentation therapy [116].

Over the past few decades, increasing attempts have been devoted to identifying effective TYR inhibitors from natural products and synthetic compounds through in vitro and in silico procedures. Generally speaking, spectrophotometry is the most commonly used in the determination of TYR activity [30,112,123]. Most inhibitors are assessed by dopachrome formation using L-tyrosine or L-DOPA as substrates. Tajima et al. synthesized a series of bibenzyl derivatives and found bibenzyl xyloside 2 to be a potent inhibitor (IC_50_ = 0.43 μM) that was 17 times more effective than kojic acid [124]. Ishioka et al. developed some novel TYR inhibitors based on the structure of rhododendron, with IC_50_ values ranging from 0.39 μM to 35.9 μM [125]. Using L-tyrosine and L-DOPA as substrates, Jung et al. designed thirteen (E)-benzylidene-1-indanone derivatives, in which BID3 was the most potent inhibitor of mushroom tyrosinase (IC_50_ = 0.034 μM, monophenolase activity; IC_50_ = 1.39 μM, diphenolase activity) [126]. Durai et al. applied evolutionary chemical binding similarity (ECBS) to screen a virtual chemical database for human TYR, which effectively identified seven potential TYR inhibitors [127]. In summary, candidate drugs with high affinity and great druggability can be rapidly identified through virtual screening in combination with the HTS methods presented in this review. All these compounds could be used as lead compounds to design novel potent TYR inhibitors for the treatment of diseases associated with TYR-overexpression [128].

## 5. Conclusions and Perspectives

Mammalian TYR catalyzes the initial and rate-limiting reactions of the melanin biosynthetic pathway, which is a relatively specific biomarker for malignant cutaneous melanoma [2,129]. Monitoring TYR activity remains significant and challenging for the discovery of novel therapeutics. In recent decades, the specific substrate-based optical method has been used for detecting TYR activity in real samples and high-throughput screening of TYR inhibitors. Herein, we reviewed the research advances of various assays, with an emphasis on their respective pros and cons. More substrate preferences and kinetic parameters were also outlined. Among them, the spectrophotometric technique is the most widely used method. Traditional assays for TYR activity mainly depend on the characteristic absorbance of colored products from the substrate L-tyrosine or L-DOPA. Recently, other means have also become more widely used, such as the introduction of nucleophiles to capture DQ to generate stable-colored adducts.

To achieve better performance in cell imaging applications, several TYR fluorescent substrates with high specificity and excellent optical properties have gradually emerged, including organic small molecules and nanocomposites. Owing to multiple advantages, such as superior selectivity, high sensitivity, and the potential for dynamic tracking, fluorescent probes could serve as versatile tools for analytical sensing and optical imaging analysis [130]. This not only facilitates the realization of high-throughput screening (HTS) of inhibitors but also evaluates the inhibitory potential of enzyme inhibitors in living cells, living tissues, and even in vivo; these findings significantly improve the efficiency and accuracy of drug discovery [131,132]. Notably, fluorescent probes-based molecular imaging can spatially localize the elevation of the TYR level (or activity) at the melanoma focus, thereby greatly reducing the risk of false-positive signals [133]. The safety of biosensors is a significant prerequisite for the biological studies of TYR in vivo. NIR probes or two-photo probes are capable of deepening photon penetration, reducing photo damage, and producing low background fluorescence, which hold great promise in biomedical imaging [134]. Furthermore, the conjugation of NIR dyes with anticancer agents assists in the synergistic management of cancer, thus integrating the merits of imaging and therapeutic effects to realize the ultimate objective of simultaneous diagnosis and treatment [135,136].

## Figures and Tables

**Table 1:** Properties of the detectable species from several substrates.

Substrates	Chemical Structures	Metabolite Stability	Coupled Reagent	Detectable Species	λ (nm)	*ε* (M^−1^·cm^−1^)	Ref.
4-Tert-butylcatechol (TBC)	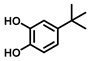	Stable	-	*o*-Quinone	400	1200	[44]
L-Tyrosine	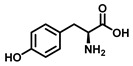	Unstable	-	Dopachrome	475	3600	[45]
L-DOPA	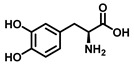	Unstable	-	Dopachrome	475	3600	[45]
			MBTH	MBTH-adduct	484	22,300	[42]
Dopamine (DA)	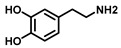	Unstable	MBTH	MBTH-adduct	503	42,500	[42]
Isoproterenol (ISO)	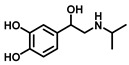	Unstable	MBTH	MBTH-adduct	497	31,500	[42]

**Table 2:** Non-fluorescent substrates of TYR.

Substrates	Chemical Structures	Enzyme Sources	Metabolites	Enzyme Activity	*K_m_* (μM)	*V_max_* (nmol/min/mg)	Ref.
L-Tyrosine	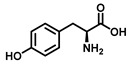	Mushroom	L-DOPA	Monophenolase activity	270	-	[41]
L-DOPA	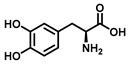	Mushroom	Dopachrome	Diphenolase activity	800	-	[41]
3-Hydroxyanthranilic acid	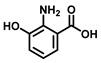	Mushroom	Cinnabarinic acid	Diphenolase activity	780	12	[61]
4-Tertbutyl-catechol	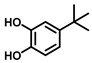	Mushroom	4-Tert-butyl-1,2-benzoquinone	Diphenolase activity	990	-	[37]

**Table 3:** Fluorescent substrates of TYR.

Names	Chemical Structures	λ_ex/em_ (nm)	Folds	*K_m_* (μM)	*V_max_* (μM·min^−1^)	LOD (U·mL^−1^)	Biological Applications	Ref.
L3	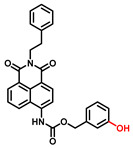	425/467 425/535	-	43.5	1.87	0.2	A375 cells	[81]
Probe 1	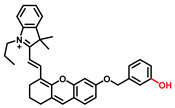	670/708	-	156	4.58	2.76	B16 and HeLa cells, zebrafish	[82]
HB-NP	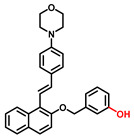	480/675	48	87.35	1.07	0.5	B16, HepG2, A549, HeLa, CCC-HPF-1 and CCC-HSF-1 cells; inhibitor screening.	[80]
Probe 1	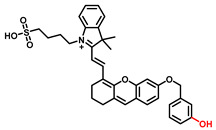	670/708	-	-	-	0.11	Imaging of TYR in B16 cells and zebrafish, melanoma diagnosis in a mouse.	[83]
Probe 1	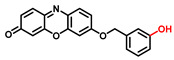	550/586	-	30	0.72	0.04	B16, HepG2 and MCF-7 cells	[84]
Probe 1	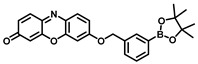	550/583	-	6.5	0.0009	0.5	B16 and HepG2 cells	[85]
NHU	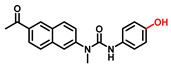	382/503	12	-	-	-	B16-F1 and HeLa cells	[86]
Probe 1	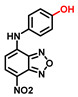	460/540	12	-	-	-	Screening inhibitors	[87]
NBR-AP	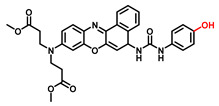	580/660	7	229.5	5.75	-	Imaging of TYR in B16F10 and HeLa cells, in vivo imaging of zebrafish and mice	[88]
Mela-TYR	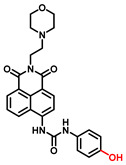	425/547	50	-	-	0.07	B16 and HeLa cells, the subcellular localization	[89]
Cy-tyr	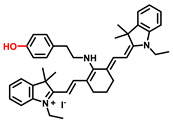	630/760 516/556	-	-	-	0.02	B16, HeLa, MCF-7 and HUVEC cells	[90]
Probe 1	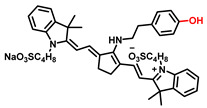	650/720	-	-	-	0.01	Screening inhibitors	[91]
MB1	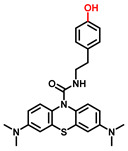	640/706	>100	4.6	0.45	-	B16F10 and HeLa cells, photodynamic therapy	[92]
Tyro-1	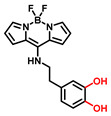	400/452	12.5	-	-	0.025	B16F10 and HeLa cells	[93]
Probe 1	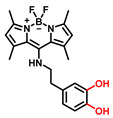	460/515	-	5.3	6.36	-	B16F10 cells	[94]
CHMC-DOPA	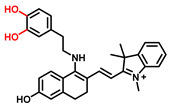	538/629	-	-	-	0.003	HepG2 cells, zebrafish	[95]

**Table 4:** The commonly used positive inhibitors and their adverse effects.

Inhibitors	Chemical Structures	Sources	Adverse Effects	Dosage	Group	Ref.
Hydroquinone (HQ)	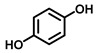	Plant	(1) Irritant contact dermatitis. (2) Exogenous ochronosis. (3) Transient erythema.	<4%	Forbidden	[117,118]
β-Arbutin	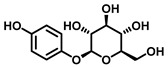	Plant	Facial tingling	<3%	Approved	[119,120]
Kojic acid (KA)	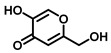	Fungus	(1) Contact dermatitis (especially in sensitive skins). (2) Long-term use may make the skin more prone to sunburn. (3) Using KA on damaged or broken skins can lead to cancer.	<1%	Approved	[121,122]

## Data Availability

Not applicable.
